# Supplementary material for: Adding salt to foods increases the risk of metabolic dysfunction-associated steatotic liver disease
Source: Commun Med (Lond). 2025 Aug 8;5:342. doi: 10.1038/s43856-025-01074-4 (PMC12334587; doi:10.1038/s43856-025-01074-4)
Supplement: Supplementary file 2 — Supplementary Information [file 43856_2025_1074_MOESM2_ESM.pdf]

Supplementary Materials

Supplementary Fig. 1. Flow chart of the study.
Supplementary Fig. 2. A Priori defined directed acyclic graph guiding the analyses.

Supplementary Table 1. ICD-10 codes used to exclude other liver diseases or alcohol/drug use disorder at/before baseline.
Supplementary Table 2. Codes used for covariates in the study.
Supplementary Table 3. Blood biomarkers used in the study.
Supplementary Table 4. Baseline characteristics of participants with available estimated 24-h urinary sodium excretion or liver PDFF data.
Supplementary Table 5. The sensitivity analysis for associations between the frequency of adding salt to foods and risk of MASLD.
Supplementary Table 6. Association between estimated 24-h urinary sodium excretion and risk of MASLD.
Supplementary Table 7. Association between frequency of adding salt to foods, PDFF and PDFF-defined MASLD.
Supplementary Table 8. Association between frequency of adding salt to foods and FIB-4 index in individuals with MASLD at baseline.
Supplementary Table 9. Spearman correlations that demonstrate consistency across multiple assessments of the frequency of adding salt to foods.

Supplementary Fig. 1. Flow chart of the study.

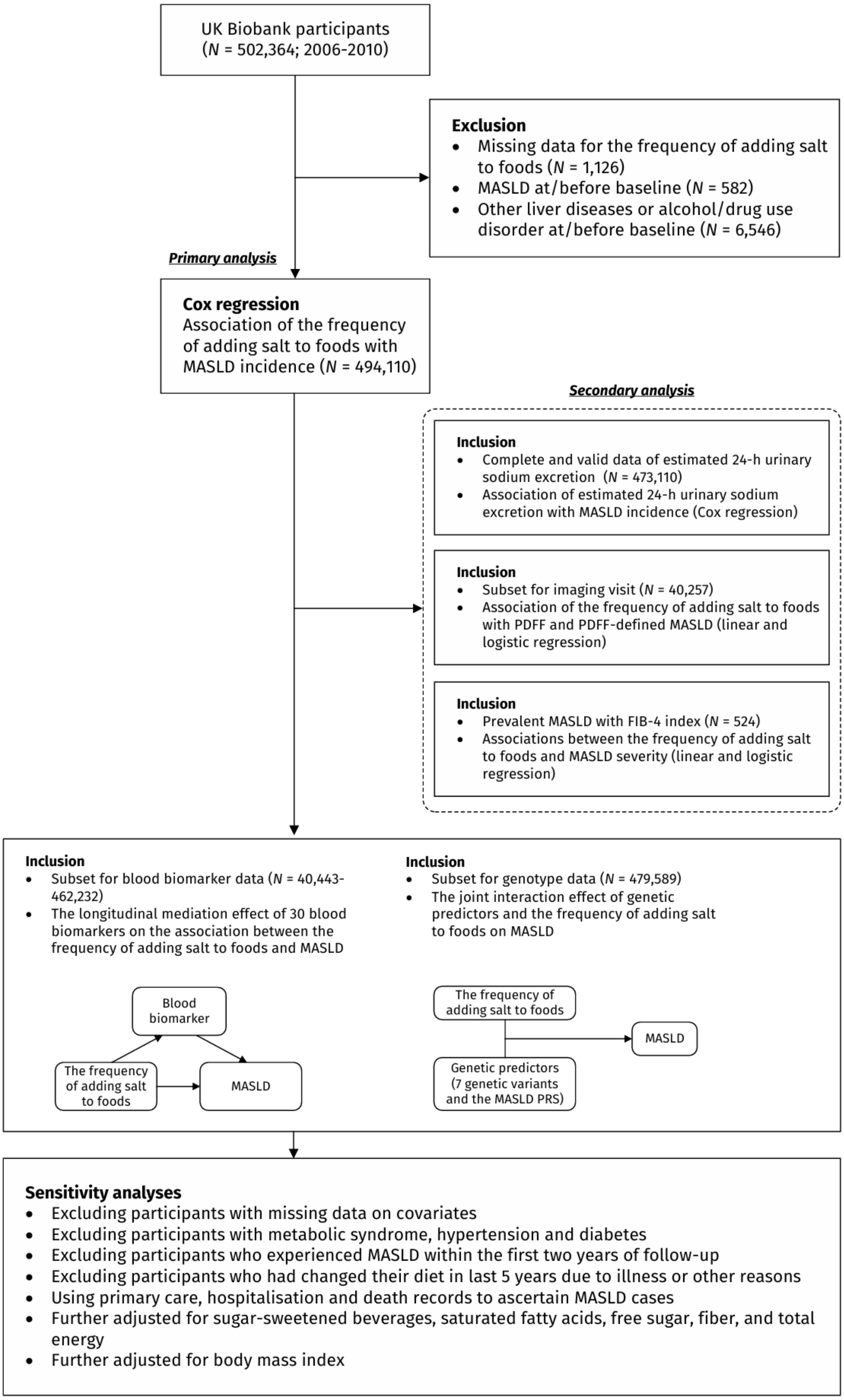

Abbreviation: *FIB-4* Fibrosis-4, *MASLD* metabolic dysfunction-associated steatotic liver disease, *PDFF* proton density fat fraction, *PRS* polygenic risk score

Supplementary Fig. 2. A Priori defined directed acyclic graph guiding the analyses.

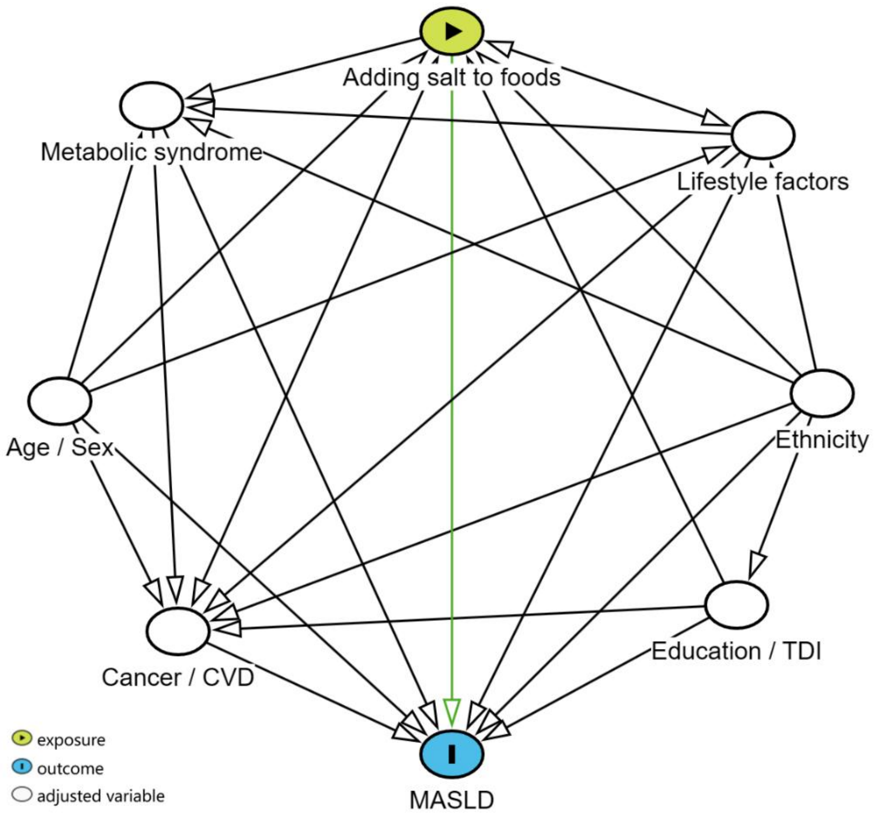

Lifestyle factors included smoking status, alcohol consumption, physical activity level, and healthy diet score.

Abbreviation: *CVD* cardiovascular disease, *MASLD* metabolic dysfunction-associated steatotic liver disease, *TDI* Townsend deprivation index

Supplementary Table 1. ICD-10 codes used to exclude other liver diseases or alcohol/drug use disorder at/before baseline.

Diagnosis	ICD-10
**Other liver diseases**	
Alcohol-associated liver disease (ALD)	K70
Viral hepatitis	B16, B17, B18, B19
Autoimmune liver disease (AIH, PBC, PSC)	K83.0A, K83.0F, K74.3, K75.4
Hemochromatosis	E83.1
Wilson's disease	E83.0
Alpha-1-antitrypsin deficiency	E88.0
Budd-Chiari syndrome	I82.0, K76.5
Chronic hepatitis, unspecified	K73.9, K73.2
Secondary or unspecified biliary cirrhosis	K74.4, K74.5
**Alcohol/drug use disorder**	
Codes associated with alcohol use disorder	F10
Codes associated with somatic consequences of alcohol (except ALD)	E24.4, G62.1, I42.6, K29.2, G31.2, G72.1, K85.2, K86.0, T51.0, T51.9, Y57.3, X65, Z50.2, Z71.4, Z72.1
Codes associated with drug use disorders except nicotine/caffeine	F11-F14, F16, F18, F19

Abbreviation: *AIH* autoimmune hepatitis, *PBC* primary biliary cholangitis, *PSC* primary sclerosing cholangitis

Supplementary Table 2. Codes used for covariates in the study.

Covariates	Field ID (baseline assessment)	ICD-10*
**Used for main analyses**		
Age at baseline assessment	21003	-
Sex	31	-
Ethnicity	21000	-
Townsend deprivation index	22189	-
Education level	6138	-
Healthy diet score	1289, 1299, 1309, 1319, 1329, 1339, 1349, 1369, 1379, 1389, 1438, 1448, 1458, 1468	-
Smoking status	20116	-
Alcohol consumption	1558	-
Physical activity level	22032	-
Metabolic syndrome severity	4080, 4079, 48, 6150, 30870, 6177, 6153, 30740, 2443	-
Cancer	2453	C00-C97
Cardiovascular disease	-	I00-I99
**Used for sensitivity analyses**		
Hypertension	6150	I10
Diabetes	2443	E10-E14
Major dietary changes in the last 5 years	1538	-
Sugar-sweetened beverages	26127#	-
Saturated fatty acids	26014#	-
Free sugar	26012#	-
Fiber	26017#	-
Total energy	26002#	-
Body mass index	21001	-

*ICD-10 codes were used to ascertain cases at/before baseline assessment in the hospital inpatient data. #Intake of nutrients and food groups were averaged across the five rounds of 24-hour dietary questionnaires.

Supplementary Table 3. Blood biomarkers used in the study.

Variables	Field ID (baseline assessment)	No. of participants
**Glucose homeostasis**		
Hemoglobin A1c (HbA1c)	30750	459170
Glucose	30740	422813
Insulin-like growth factor-1 (IGF-1)	30770	459703
**Inflammation**		
C-reactive protein (CRP)	30710	461223
Rheumatoid factor	30820	40443
**Kidney function**		
Calcium	30680	423174
Creatinine	30700	461986
Cystatin C	30720	462184
Phosphate	30810	422509
Urate	30880	461659
Urea	30670	461905
Vitamin D	30890	441398
**Lipid metabolism**		
Apolipoprotein A	30630	420829
Apolipoprotein B	30640	459890
High-density lipoprotein cholesterol (HDL-C)	30760	423121
Lipoprotein A	30790	369881
Low-density lipoprotein direct (LDL-C)	30780	461351
Total cholesterol	30690	462217
Triglycerides	30870	461855
**Liver function**		
Alanine aminotransferase (ALT)	30620	462036
Albumin	30600	423316
Alkaline phosphatase (ALP)	30610	462232
Aspartate aminotransferase (AST)	30650	460462
Direct bilirubin	30660	392199
Total bilirubin	30840	460228
Gamma glutamyltransferase (GGT)	30730	461994
Total protein	30860	422852
Sex hormone-binding globulin (SHBG)	30830	419154
Oestradiol	30800	75456
Testosterone	30850	418385

Supplementary Table 4. Baseline characteristics of participants with available estimated 24-h urinary sodium excretion or liver PDFF data.

Characteristic	All participants (*N* = 494,110)	Participants with estimated 24-h urinary sodium excretion data (*N* = 473,110)	Participants with liver PDFF data (*N* = 40,257)
Age, years	56.5 (8.1)	56.5 (8.1)	55.0 (7.5)
Sex			
Female	270,420 (54.7%)	258,124 (54.6%)	21,065 (52.3%)
Male	223,690 (45.3%)	214,976 (45.4%)	19,192 (47.7%)
Ethnicity			
Non-White	26,769 (5.4%)	25,001 (5.3%)	1,218 (3.0%)
White	467,341 (94.6%)	448,099 (94.7%)	39,039 (97.0%)
Townsend deprivation index	-1.3 (3.1)	-1.4 (3.1)	-1.9 (2.7)
Education level			
Less than high school	84,957 (17.2%)	80,836 (17.1%)	2,562 (6.4%)
High school or equivalent	246,709 (49.9%)	236,434 (50.0%)	18,766 (46.6%)
College or above	162,444 (32.9%)	155,830 (32.9%)	18,929 (47.0%)
Healthy diet score	3.6 (1.5)	3.6 (1.5)	3.7 (1.5)
Smoking status			
Never	272,264 (55.1%)	261,002 (55.2%)	24,571 (61.0%)
Previous	171,077 (34.6%)	164,022 (34.7%)	13,230 (32.9%)
Current	50,769 (10.3%)	48,076 (10.2%)	2,456 (6.1%)
Alcohol consumption			
None currently	39,447 (8.0%)	37,031 (7.8%)	1,854 (4.6%)
Up to twice per week	240,663 (48.7%)	230,397 (48.7%)	17,947 (44.6%)
Three or more times per week	214,000 (43.3%)	205,672 (43.5%)	20,456 (50.8%)
Physical activity level			
Low	92,555 (18.7%)	87,843 (18.6%)	7,342 (18.2%)
Moderate	200,323 (40.5%)	192,058 (40.6%)	16,869 (41.9%)
High	201,232 (40.7%)	193,199 (40.8%)	16,046 (39.9%)
Metabolic syndrome severity	2.2 (1.3)	2.2 (1.3)	1.9 (1.3)
Cancer	41,442 (8.4)	39,597 (8.4)	2,423 (6.0)
Cardiovascular disease	78,071 (15.8)	74,056 (15.7)	4,265 (10.6)
Frequency of adding salt to foods			
Never/rarely	274,781 (55.6%)	263,176 (55.6%)	24,218 (60.2%)
Sometimes	138,627 (28.1%)	132,925 (28.1%)	10,675 (26.5%)
Usually	57,190 (11.6%)	54,693 (11.6%)	4,167 (10.4%)
Always	23,512 (4.8%)	22,306 (4.7%)	1,197 (3.0%)
Estimated 24-h urinary sodium excretion, g	-	3.0 (0.8)	-
PDFF, %	-	-	4.9 (4.9)
PDFF-defined MASLD	-	-	9,709 (24.1)

Data are n (%) or mean (SD).

Abbreviation: *MASLD* metabolic dysfunction-associated steatotic liver disease, *PDFF* proton density fat fraction

Supplementary Table 5. The sensitivity analysis for associations between the frequency of adding salt to foods and risk of MASLD.

**Sensitivity analysis 1: excluding participants with missing data on covariates**					
	Never/rarely	Sometimes	Usually	Always	*P* for trend
Events No./total No.	2056/171719	1125/82832	564/34286	261/12834	
Multivariable adjusted HR (95% CI)	1 (reference)	1.06 (0.98-1.14)	1.23 (1.12-1.36)	1.30 (1.14-1.48)	1.89E-07
**Sensitivity analysis 2: excluding participants with metabolic syndrome, hypertension and diabetes***					
	Never/rarely	Sometimes	Usually	Always	*P* for trend
Events No./total No.	1323/170592	773/87906	386/36243	209/14718	
Multivariable adjusted HR (95% CI)	1 (reference)	1.05 (0.96-1.15)	1.21 (1.08-1.35)	1.36 (1.17-1.58)	4.72E-06
**Sensitivity analysis 3: excluding participants who experienced MASLD within the first two years of follow-up**					
	Never/rarely	Sometimes	Usually	Always	*P* for trend
Events No./total No.	3285/274649	1898/138552	916/57159	490/23488	
Multivariable adjusted HR (95% CI)	1 (reference)	1.07 (1.02-1.14)	1.20 (1.12-1.29)	1.34 (1.22-1.47)	3.89E-08
**Sensitivity analysis 4: excluding participants who had changed their diet in last 5 years due to illness or other reasons**					
	Never/rarely	Sometimes	Usually	Always	*P* for trend
Events No./total No.	1625/165306	927/83928	491/35603	291/14948	
Multivariable adjusted HR (95% CI)	1 (reference)	1.03 (0.95-1.12)	1.19 (1.08-1.32)	1.42 (1.25-1.62)	2.11E-12
**Sensitivity analysis 5: using primary care, hospitalisation and death records to ascertain MASLD cases**					
	Never/rarely	Sometimes	Usually	Always	*P* for trend
Events No./total No.	3568/274773	2067/138621	984/57189	536/23511	
Multivariable adjusted HR (95% CI)	1 (reference)	1.07 (1.02-1.13)	1.20 (1.11-1.29)	1.35 (1.23-1.48)	3.98E-13
**Sensitivity analysis 6: further adjusted for sugar-sweetened beverages, saturated fatty acids, free sugar, fiber, and total energy**					
	Never/rarely	Sometimes	Usually	Always	*P* for trend
Events No./total No.	1242/122815	690/56464	346/22284	130/7117	
Multivariable adjusted HR (95% CI)	1 (reference)	1.13 (1.03-1.24)	1.35 (1.20-1.53)	1.37 (1.14-1.64)	4.44E-08
**Sensitivity analysis 7: further adjusted for body mass index**					
	Never/rarely	Sometimes	Usually	Always	*P* for trend
Events No./total No.	3550/273480	2056/137893	972/56807	530/23307	
Multivariable adjusted HR (95% CI)	1 (reference)	1.04 (0.98-1.10)	1.14 (1.06-1.23)	1.31 (1.19-1.44)	3.79E-09

Results were adjusted for age, sex, ethnicity, Townsend deprivation index, education level, healthy diet score, smoking status, alcohol consumption, physical activity level, metabolic syndrome severity, cancer, and cardiovascular disease. *Metabolic syndrome was defined as metabolic syndrome severity > 3.

Supplementary Table 6. Association between estimated 24-h urinary sodium excretion and risk of MASLD.

	HR (95% CI)	
	**Model 1**	**Model 2**
**Estimated 24-hour urinary sodium excretion**		
Quartile 1 (0 to 2.37 g)	1 (reference)	1 (reference)
Quartile 2 (2.37 to 2.86 g)	1.67 (1.54-1.82)	1.36 (1.25-1.47)
Quartile 3 (2.86 to 3.53 g)	3.03 (2.78-3.30)	1.89 (1.73-2.06)
Quartile 4 (3.53 to 9.08 g)	5.63 (5.10-6.20)	2.61 (2.36-2.88)
***P* for trend**	1E-350	2.05E-104

Abbreviation: *MASLD* metabolic dysfunction-associated steatotic liver disease.

Model 1: Adjusted for age, sex

Model 2: Model 1 + ethnicity, Townsend deprivation index, education level, healthy diet score, smoking status, alcohol consumption, physical activity level, metabolic syndrome severity, cancer, and cardiovascular disease

Supplementary Table 7. Association between frequency of adding salt to foods, PDFF and PDFF-defined MASLD.

	Frequency of adding salt to foods, β (95% CI)				
	Never/rarely	Sometimes	Usually	Always	*P* for trend
**PDFF**					
No. of participants	24218	10675	4167	1197	
Model 1	0 (reference)	0.47 (0.36-0.58)	0.55 (0.39-0.71)	0.76 (0.48-1.04)	2.63E-23
Model 2	0 (reference)	0.31 (0.21-0.42)	0.32 (0.17-0.47)	0.31 (0.04-0.58)	8.22E-09
	**Frequency of adding salt to foods, OR (95% CI)**				
	Never/rarely	Sometimes	Usually	Always	*P* for trend
**PDFF-defined MASLD**					
Events No./total No.	5407/24218	2801/10675	1153/4167	348/1197	
Model 1	1 (reference)	1.23 (1.17-1.30)	1.29 (1.20-1.39)	1.45 (1.27-1.65)	1.94E-22
Model 2	1 (reference)	1.17 (1.10-1.23)	1.18 (1.09-1.28)	1.19 (1.03-1.36)	3.59E-08

Abbreviation: *MASLD* metabolic dysfunction-associated steatotic liver disease, *PDFF* proton density fat fraction.

Model 1: Adjusted for age, sex

Model 2: Model 1 + ethnicity, Townsend deprivation index, education level, healthy diet score, smoking status, alcohol consumption, physical activity level, metabolic syndrome severity, cancer, and cardiovascular disease

Supplementary Table 8. Association between frequency of adding salt to foods and FIB-4 index in individuals with MASLD at baseline.

	**Frequency of adding salt to foods, β (95% CI)**				
	Never/rarely	Sometimes	Usually	Always	*P* for trend
**FIB-4 (continuous)**					
No. of participants	251	147	84	42	
Model 1	0 (reference)	0.15 (-0.06, 0.35)	-0.07 (-0.32, 0.19)	0.39 (0.06, 0.72)	0.15
Model 2	0 (reference)	0.16 (-0.05, 0.37)	-0.07 (-0.32, 0.19)	0.37 (0.03, 0.70)	0.19
	**Frequency of adding salt to foods, OR (95% CI)**				
	Never/rarely	Sometimes	Usually	Always	*P* for trend
**FIB-4 (binary)**					
Events No./total No.	19/251	13/147	8/84	9/42	
Model 1	1 (reference)	1.21 (0.56-2.53)	1.11 (0.44-2.60)	3.75 (1.47-9.08)	0.03
Model 2	1 (reference)	1.29 (0.59-2.74)	1.09 (0.42-2.60)	3.55 (1.36-8.85)	0.04

Abbreviation: *MASLD* metabolic dysfunction-associated steatotic liver disease, *FIB-4* Fibrosis-4.

We classified participants with FIB-4 index > 2.67 as having a high risk of advanced fibrosis, and those with FIB-4 ≤ 2.67 as having a low/indeterminate risk of liver fibrosis. We used FIB-4 ≤ 2.67 as reference.

Model 1: Adjusted for age, sex

Model 2: Model 1 + ethnicity, Townsend deprivation index, education level, healthy diet score, smoking status, alcohol consumption, physical activity level, metabolic syndrome severity, cancer, and cardiovascular disease

Supplementary Table 9. Spearman correlations that demonstrate consistency across multiple assessments of the frequency of adding salt to foods.

	Baseline assessment (2006-2010)	First repeat assessment visit (2012-2013)	Imaging visit (2014+)	First repeat imaging visit (2019+)
Baseline assessment (2006-2010)	1	**0.68 (*n*=20328)**	**0.60 (*n*=69833)**	**0.59 (*n*=5996)**
First repeat assessment visit (2012-2013)	-	1	**0.68 (*n*=9544)**	**0.65 (*n*=931)**
Imaging visit (2014+)	-	-	1	**0.70 (*n*=5966)**
First repeat imaging visit (2019+)	-	-	-	1
