# Supplementary material for: Adding salt to foods increases the risk of metabolic dysfunction-associated steatotic liver disease
Source: Commun Med (Lond). 2025 Aug 8;5:342. doi: 10.1038/s43856-025-01074-4 (PMC12334587; doi:10.1038/s43856-025-01074-4)
Supplement: Supplementary file 3 — Description of Additional Supplementary files [file 43856_2025_1074_MOESM3_ESM.pdf]

Description of Additional Supplementary files

File name: Supplementary Data 1

Description: Stratified analyses for association between the frequency of adding salt to foods and risk of MASLD.

File name: Supplementary Data 2

Description: Mediation effect of 30 blood biomarkers on the relationship between the frequency of adding salt to foods (reference group: never/rarely) and risk of MASLD.

File name: Supplementary Data 3

Description: Joint associations between genetic predictors and the frequency of adding salt to foods on the risk of MASLD.
