# Supplementary material for: Adding salt to foods increases the risk of metabolic dysfunction-associated steatotic liver disease
Source: Commun Med (Lond). 2025 Aug 8;5:342. doi: 10.1038/s43856-025-01074-4 (PMC12334587; doi:10.1038/s43856-025-01074-4)
Supplement: Supplementary file 7 — Reporting summary [file 43856_2025_1074_MOESM7_ESM.pdf]

nature portfolio

Corresponding author(s): Qiong Wu
Shujuan Lin

Last updated by author(s): Jul 13, 2025

Reporting Summary

Nature Portfolio wishes to improve the reproducibility of the work that we publish. This form provides structure for consistency and transparency in reporting. For further information on Nature Portfolio policies, see our Editorial Policies and the Editorial Policy Checklist.

Statistics

For all statistical analyses, confirm that the following items are present in the figure legend, table legend, main text, or Methods section.

n/a	Confirmed	
☐	☒	The exact sample size (*n*) for each experimental group/condition, given as a discrete number and unit of measurement
☒	☐	A statement on whether measurements were taken from distinct samples or whether the same sample was measured repeatedly
☐	☒	The statistical test(s) used AND whether they are one- or two-sided<br>*Only common tests should be described solely by name; describe more complex techniques in the Methods section.*
☐	☒	A description of all covariates tested
☒	☐	A description of any assumptions or corrections, such as tests of normality and adjustment for multiple comparisons
☐	☒	A full description of the statistical parameters including central tendency (e.g. means) or other basic estimates (e.g. regression coefficient) AND variation (e.g. standard deviation) or associated estimates of uncertainty (e.g. confidence intervals)
☐	☒	For null hypothesis testing, the test statistic (e.g. *F*, *t*, *r*) with confidence intervals, effect sizes, degrees of freedom and *P* value noted<br>*Give P values as exact values whenever suitable.*
☒	☐	For Bayesian analysis, information on the choice of priors and Markov chain Monte Carlo settings
☒	☐	For hierarchical and complex designs, identification of the appropriate level for tests and full reporting of outcomes
☐	☒	Estimates of effect sizes (e.g. Cohen's *d*, Pearson's *r*), indicating how they were calculated

Our web collection on statistics for biologists contains articles on many of the points above.

Software and code

Policy information about availability of computer code

Data collection	No software was used to collect data for this study. All data in this study were derived from the UK Biobank.
Data analysis	SAS version 9.4 (SAS Institute) and R version 4.3.1 was used to perform data analysis. The codes are available upon request from corresponding authors.

For manuscripts utilizing custom algorithms or software that are central to the research but not yet described in published literature, software must be made available to editors and reviewers. We strongly encourage code deposition in a community repository (e.g. GitHub). See the Nature Portfolio guidelines for submitting code & software for further information.

Data

Policy information about availability of data

All manuscripts must include a data availability statement. This statement should provide the following information, where applicable:

- Accession codes, unique identifiers, or web links for publicly available datasets
- A description of any restrictions on data availability
- For clinical datasets or third party data, please ensure that the statement adheres to our policy

All UK Biobank information is available online (https://www.ukbiobank.ac.uk/), and data can be accessed upon application.

Research involving human participants, their data, or biological material

Policy information about studies with human participants or human data. See also policy information about sex, gender (identity/presentation), and sexual orientation and race, ethnicity and racism.

Reporting on sex and gender	The reported sex of participants was accessed through UK Biobank Data-Field 31, and included as a covariate in the statistical analyses. Gender was not considered in this study.
Reporting on race, ethnicity, or other socially relevant groupings	The ethnicity of participants was accessed through UK Biobank Data-Field 21000, and included as a covariate in the statistical analyses.
Population characteristics	During a median follow-up time of 13.6 years (interquartile range 12.2-15.1 years), 7,171 (1.45 %) cases of incident MASLD were recorded. Baseline characteristics of participants, categorized by the frequency of adding salt to foods, are presented in Table 1. Compared to participants with a lower frequency of adding salt to foods, those with a higher frequency were more likely to be male, to be non-White, and have a higher Townsend deprivation index and a lower level of education; These participants also tended to have a lower healthy diet score, were more likely to be current smokers, less physically active, and exhibit a higher severity of metabolic syndrome. Additionally, they had a higher prevalence of cancer and cardiovascular disease.
Recruitment	The UK Biobank is a large population-based prospective cohort study that recruited over 500,000 participants, aged between 37 and 73 years, across 22 assessment centers in England, Scotland, and Wales between 2006 and 2010. During the baseline assessment, participants completed nurse-administrated touchscreen questionnaires covering demographic characteristics and lifestyle and health-related information, underwent physical examinations, and provided biological samples for genotyping and laboratory assay. Additionally, approximately 40,000 participants were invited to undergo abdominal MRI as part of the UK Biobank's imaging enhancement project. From the initial sample of 502,364 participants, we excluded 1,708 participants with incomplete data on the frequency of adding salt to foods, and those with prevalent MASLD at baseline. An additional 6,546 participants with other liver diseases or alcohol/drug use disorder at baseline were excluded. This left 494,110 participants for the primary analysis.
Ethics oversight	UK Biobank has approval from the North West Multi-centre Research Ethics Committee (MREC) as a Research Tissue Bank (RTB) approval. This approval means that researchers do not require separate ethical clearance and can operate under the RTB approval. Informed consents were obtained from all participants. The ethical approval for the UK Biobank is extensively detailed and described online: https://www.ukbiobank.ac.uk/learn-more-about-uk-biobank/about-us/ethics. This current study was undertaken under application number 95817.

Note that full information on the approval of the study protocol must also be provided in the manuscript.

Field-specific reporting

Please select the one below that is the best fit for your research. If you are not sure, read the appropriate sections before making your selection.

☒ Life sciences ☐ Behavioural & social sciences ☐ Ecological, evolutionary & environmental sciences

For a reference copy of the document with all sections, see nature.com/documents/nr-reporting-summary-flat.pdf

Life sciences study design

All studies must disclose on these points even when the disclosure is negative.

Sample size	Our final sample consisted of N = 494,110 participants for the primary analysis.
Data exclusions	The data exclusion criteria was documented in Methods section. From the initial sample of 502,364 participants, we excluded 1,708 participants with incomplete data on the frequency of adding salt to foods, and those with prevalent MASLD at baseline. Following the latest Expert Panel Consensus Statement, an additional 6,546 participants with other liver diseases or alcohol/drug use disorder at baseline were excluded.
Replication	This is a population-based cohort study, the findings of which may need to be validated by further clinical trials and/or larger cohorts.
Randomization	This is an observational cohort study, and randomization is not applicable in this study. However, we carefully controlled for a wide range of potential covariates in the Cox regression models.
Blinding	The investigators were blinded to the exposure group during the statistical analyses for testing the exposure-outcome associations.

Reporting for specific materials, systems and methods

We require information from authors about some types of materials, experimental systems and methods used in many studies. Here, indicate whether each material, system or method listed is relevant to your study. If you are not sure if a list item applies to your research, read the appropriate section before selecting a response.

Materials & experimental systems

n/a	Involved in the study
☒	☐ Antibodies
☒	☐ Eukaryotic cell lines
☒	☐ Palaeontology and archaeology
☒	☐ Animals and other organisms
☒	☐ Clinical data
☒	☐ Dual use research of concern
☒	☐ Plants

Methods

n/a	Involved in the study
☒	☐ ChIP-seq
☒	☐ Flow cytometry
☒	☐ MRI-based neuroimaging

Plants

Seed stocks

Report on the source of all seed stocks or other plant material used. If applicable, state the seed stock centre and catalogue number. If plant specimens were collected from the field, describe the collection location, date and sampling procedures.

Novel plant genotypes

Describe the methods by which all novel plant genotypes were produced. This includes those generated by transgenic approaches, gene editing, chemical/radiation-based mutagenesis and hybridization. For transgenic lines, describe the transformation method, the number of independent lines analyzed and the generation upon which experiments were performed. For gene-edited lines, describe the editor used, the endogenous sequence targeted for editing, the targeting guide RNA sequence (if applicable) and how the editor was applied.

Authentication

Describe any authentication procedures for each seed stock used or novel genotype generated. Describe any experiments used to assess the effect of a mutation and, where applicable, how potential secondary effects (e.g. second site T-DNA insertions, mosiacism, off-target gene editing) were examined.
